# Effectiveness of binaural beat music combined with rhythmical photic stimulation on older people with depressive symptoms in long-term care institution: a quasi-experimental pilot study

**DOI:** 10.1007/s40520-024-02737-3

**Published:** 2024-04-01

**Authors:** Shang-Yu Yang, Pin-Hsuan Lin, Jiun-Yi Wang, Shih-Hau Fu

**Affiliations:** 1 Department of Healthcare Administration, College of Medical and Health Science, Asia University, 500, Lioufeng Rd, 41354 Wufeng, Taichung, Taiwan, R.O.C.; 2 Department of Health and Beauty, Shu Zen Junior College of Medicine and Management, Kaohsiung, 821 Taiwan; 3 Department of Medical Research, China Medical University Hospital, China Medical University, Taichung, 404 Taiwan; 4 Department of Acupressure Technology, Chung Hwa University of Medical Technology, Tainan 717, Tainan, Taiwan

**Keywords:** Binaural beat, Rhythmical photic stimulation, Depressive symptoms, Long-term care, Older adults

## Abstract

**Background:**

Many older adults residing in long-term care often face issues like poor sleep, reduced vitality, and depression. Non-pharmacological approaches, specifically Binaural Beat Music (BBM) and Rhythmic Photic Stimulation (RPS), may alleviate these symptoms, yet their efficacy in this demographic has not been extensively explored.

**Aims:**

This study investigated the effects of combined BBM and RPS interventions on sleep quality, vitality, and depression among older residents with depressive symptoms in long-term care facilities.

**Methods:**

Using a quasi-experimental design, a total of 88 older adults with depressive symptoms from Taiwanese daytime care centers were divided into the BBM with RPS, and Sham groups (44 each). They underwent 20-minute daily sessions of their assigned treatment for two weeks. The BBM with RPS group listened to 10 Hz binaural beat music with 10 Hz photic stimulation glasses, and the Sham group received non-stimulating music and glasses.

**Results:**

After the intervention, participants in the BBM with RPS groups showed significant improvements in vitality and depressive mood, with a notable increase in sympathetic nervous system activity. Conversely, the Sham group exhibited significant deterioration in vitality and mental health, with a significant increase in parasympathetic activity. Additionally, compared with the Sham group, the BBM and RPS groups showed significant improvements in vitality, mental health, and depression, with a significant increase in sympathetic nervous activity.

**Conclusion:**

The two-week intervention suggests that the combination of BBM and RPS, as a non-invasive intervention, can potentially improve vitality, mental health, and depressive mood among older adults in long-term care institutions.

## Introduction

Compared to older adults living in the community, residents of long-term care facilities commonly face sleep quality issues [1], including nighttime sleep interruptions and increased daytime fatigue [2]. Poor sleep quality directly affects physical health by increasing the risk of nighttime falls and exacerbating chronic disease symptoms [3], and can even lead to higher mortality rates [4]. It also leads to decreased daytime vitality, potentially causing long-term adverse effects on physical and mental health such as reduced physical and social activities, diminished self-care abilities, depression, and anxiety [3, 5–7]. Hence, many studies have focused on non-invasive, non-pharmacological interventions to improve sleep quality in older adults [8].

Older residents in long-term care facilities often exhibit a higher tendency toward depression [9]. Previous studies [10] have shown that the prevalence of depression among these residents ranges from 31.3 to 94.2%, compared with 6–23.3% in community-dwelling adults. Depressive symptoms are associated with a decline in cognitive function, social isolation, and worsening physical health [11, 12]. Depression in older adults can manifest as loss of appetite, weight loss, sleep disturbances, and reduced activity, further exacerbating their health conditions [12–14]. Moreover, depressive symptoms can increase the risk of suicide among older adults, especially in the absence of adequate social and mental health support [12, 14, 15]. This highlights the need for long-term care facilities to pay attention to the mental health of older residents and to find effective prevention and treatment methods.

Binaural Beat Music (BBM) is a non-invasive non-pharmacological type of music therapy that offers a unique approach to mental and emotional well-being. It works by playing two slightly different frequency tones, one to each ear. The brain then perceives this difference as a single beat, known as a binaural beat, which is thought to entrain or synchronize brain waves to the frequency of the perceived beat. Previous research [16] suggests that music interventions, including BBM, can positively impact sleep quality in older adults, particularly by reducing sleep latency, prolonging sleep duration, enhancing sleep efficiency, and improving daytime dysfunction. BBM may enhance daytime vitality in older adults by improving their sleep quality. Sung et al. [17] also found that listening to a 10 Hz BBM for 30 min daily for five days significantly reduced depression levels among older adults in long-term care. Furthermore, da Silva Junior et al. [18] and Yusim, Grigaitis [19] indicated that listening to BBM helps reduce anxiety and improve mood.

Rhythmic Photic Stimulation (RPS) has become an increasingly popular neuroscientific and psychological technique. This technique involves the presentation of repetitive flashes of light to the visual system, aiming to induce changes in brainwave patterns and modulate brain activity. This technique is widely used to study the brain’s response to light and is gradually being used to treat emotional disorders, improve cognition, and enhance mental health [20, 21]. Rhythmic photo stimulation (RPS) operates on the principle that the brain’s blood flow responds to the frequency of light exposure. Low-frequency stimulation, in particular, enhances cerebral blood flow in areas associated with vision, making it valuable for promoting deep relaxation and meditation [22]. Previous studies have found that RPS affects biomarkers such as neurotransmitters. For instance, a study by Shealy et al. [23] found that a 30-minute session of 10 Hz light stimulation increased serotonin and endorphin levels in patients with anxiety disorders, while simultaneously lowering daytime melatonin levels. While these findings may be beneficial for improving sleep quality, daytime activities, and depressive mood (e.g., serotonin regulation in depression), further research is required to confirm their effectiveness.

The combination of auditory and visual stimuli has made significant progress over the past decade, mainly owing to more precise control over light stimulation [22]. Related equipment is increasingly used by households and therapists, lasting from 20-60-minute sessions in seated or lying positions [22, 24]. Many studies [22, 25, 26] have demonstrated the benefits of combining BBM and RPS in providing low-frequency audiovisual stimulation (e.g., 10 Hz) to initiate and maintain sleep. However, the effectiveness of audiovisual stimulation in addressing sleep, vitality, and mental health issues among older residents of long-term care facilities remains unclear. Therefore, this study aimed to explore the effects of combined BBM and RPS interventions on older residents with depressive symptoms in long-term care facilities, focusing on sleep quality, vitality, and depression.

## Methods

### Study design and participants

This quasi-experimental study was conducted at two daytime care centers in central Taiwan. Participants were not randomly assigned to groups but were instead grouped based on their respective care centers. Residents of one center formed the experimental groups (BBM and RPS groups), while residents of the other center formed the control group (Sham group). The recruitment period was from June to August 2023. The intervention lasted for 14 days, with participants undergoing pre-testing two days prior. This pre-testing, conducted by the researchers, included questionnaire interviews and heart rate variability (HRV) analysis. The questionnaires assessed demographic information, sleep quality using the Pittsburgh Sleep Quality Index (PSQI), vitality using the Vitality Scale (VT), mental health using the Mental Health Scale (MH), and depression using the Hamilton Depression Rating Scale (HAMD). HRV was assessed using an HRV analyzer. Post-testing, identical to pre-testing, was conducted after the 14th day of intervention.

The eligibility criteria for the study were as follows: (1) age 65 years or above; (2) HAMD score > 7 (indicative of depressive symptoms); (3) ability to understand the questionnaire content; (4) ability to communicate in Mandarin or Taiwanese; (5) residence in the institution for at least one month; and (6) no antidepressant medication use within three months before or during the intervention. The exclusion criteria were as follows: (1) hearing impairment; (2) diagnosis of bipolar disorder or schizophrenia; and (3) acute physical illnesses, such as colds, fractures, or injuries.

The sample size was determined using a G-power software (version 3.1.9) based on data from our preliminary study. This pilot study, involving 25 participants in each group, showed a mean change in nHF (high-frequency normalized units) in HRV of -0.97/0.91 and − 0.22/0.61 in the experimental and control groups, respectively. With a sample of 25 participants per group, the study achieved 95% statistical power and an alpha level of 0.05, sufficient to detect significant difference between groups. A total of 88 participants (44 in each group) were recruited for this study, and all successfully completed the 14-day post-test without any dropouts. This study was approved by the Research Ethics Committee of China Medical University Hospital (CMUH110-REC3-021).

### Intervention procedures

All participants underwent a 20-minute intervention every weekday for two consecutive weeks. Before the intervention, the participants completed questionnaire interviews and HRV analysis as pre-tests. The intervention involved resting in a chair for 10 min, followed by a 20-minute session with their eyes closed. All sessions were conducted between 9 AM and 11 AM in a quiet room at a moderate temperature. Post-tests, identical to pre-tests, were conducted within two days of the 2-week intervention.

In the BBM and RPS groups, participants were equipped with over-ear stereo headphones and RPS glasses and to receive 20 min of 10 Hz BBM (embedded within relaxing music as a masking element) and RPS. The BBM and RPS glasses were provided by George Szeless (MindLightz Software). Participants in the Sham group wore identical equipment but received only relaxing music without the 10 Hz stimulation in the RPS glasses. To ensure the participants’ comfort and prevent adverse reactions, research assistants commenced data checks after the first 5 min of the intervention.

### Measurements

Questionnaire measures included demographic information, PSQI, Vitality Index, Mental Health scale, and HAMD, whereas instrument measures included HRV analysis. Demographic data included sex, age, marital status, education level, number of chronic diseases, self-perceived health status, and disability level (light to severe: levels 1 to 6). Sleep quality was assessed using the PSQI, which includes seven components, with a total score ranging from 0 to 21. Higher PSQI scores indicated worse sleep quality. The Taiwanese version of the PSQI is known for its reliability and validity in assessing sleep quality within the Taiwanese population [27].

The Vitality Index and Mental Health scale were derived from the SF-36 health survey’s vitality and mental health subscales (of). The Taiwanese version of the SF-36, which covers eight domains with 36 items, each assessable independently, is recognized for its excellent reliability and validity within the Taiwanese context [28]. . The VT has four items (score range 4–24) and the MH has five items (score range 5–30), with higher scores indicating better vitality or mental health.

Depression levels were measured using the HAMD, a 17-item scale with a scoring range of 0–50, with higher scores indicating more severe depression. The HAMD assessment, conducted by psychologists at care centers, utilizes the Taiwanese version, which is known for its good reliability and validity, ensuring its appropriateness and accuracy for the Taiwanese population [29].

HRV was measured using an HRV SA-3000p analyzer (Medicore Co., Ltd., Seoul, Korea) to assess the mean heart rate (MHR), SDNN, nLF, nHF, and LF/HF ratio. A higher MHR indicates a faster heartbeat, a higher SDNN indicates better autonomic nervous regulation, a higher nLF indicates a greater contribution of sympathetic nerves, a higher nHF indicates a greater contribution of parasympathetic nerves, and the LF/HF ratio reflects the balance between the sympathetic and parasympathetic nerves.

### Data analysis

Data analysis was conducted using SPSS software (version 22.0; IBM Corp., Armonk, NY, USA). Descriptive statistics were used to present the demographic characteristics of the participants, and Fisher’s Exact Test was used to assess significant differences between the groups. Paired t-tests were performed to compare pre-and post-test scores on the PSQI, VT, MH, HAMD, and HRV parameters (all normally distributed). ANCOVA, adjusted for demographic variables with significant differences between groups, examined changes in the four scales and five HRV parameters (“Posttest” minus “Pretest”). The significance level was set at *p* = 0.05.

## Results

### Participants

The study recruited 88 participants (44 in each group). There were 28 males and 60 females, with an average age of 79.66 ± 7.21 years. The basic participant information and Fisher’s Exact Test results are presented in Table 1. The Fisher’s Exact Test revealed a significant difference in the level of disability between the groups, with the BBM and RPS groups having a higher level of disability.

**Table 1:** Demographic characteristics of participants in the two groups

Demographic characteristics	BBM and RPS group (*n* = 44)	Sham group (*n* = 44)	Fisher’s exact test (*p*-value)
Gender			0.11
Male	10	18	
Female	34	26	
Age (mean ± SD)	79.95 ± 6.05	79.36 ± 8.28	0.70^a^
Marital Status			0.28
Single/Divorced/Widowed	22	28	
Married/Cohabiting	22	16	
Years of education			0.79
Elementary school or below	36	34	
Junior high school or above	8	10	
Number of chronic diseases	1.41 ± 1.42	1.14 ± 0.88	0.28^a^
Self-Perceived Health Status			0.33
Very good	4	8	
Good	14	16	
Fair	20	18	
Poor	6	2	
Level of disability	3.05 ± 0.78	2.55 ± 0.59	< 0.01*

^a^ Student’s t-test; *: *p* < 0.05

### Changes in PSQI, VT, MH, HAMD, and HRV parameters before and after intervention

The average values, standard deviations, and paired t-test results for the PSQI, VT, MH, HAMD, and HRV parameters are presented in Table 2. In the BM and RPS groups, significant differences were observed in the VT and HAMD pre- and post-tests (*p* < 0.05), indicating significant improvements in vitality and depression levels after the intervention. Furthermore, for the HRV results, significant differences in nHF (*p* < 0.05) suggested increased sympathetic nerve activation after the intervention.

**Table 2:** Changes in PSQI, VT, MH, HAMD, and HRV parameters before and after intervention

	BBM and RPS group (*n* = 44)			Sham group (*n* = 44)		
	Pre-test	Post-test		Pre-test	Post-test	
Outcome variable	Mean (SD)	Mean (SD)	*p*-value	Mean (SD)	Mean (SD)	*p*-value
PSQI	11.09 (4.67)	11.17 (3.90)	0.40	7.36 (1.99)	7.23 (2.07)	0.66
VT	13.95 (3.72)	15.23 (2.78)	0.02*	18.50 (2.04)	17.41 (1.52)	< 0.01*
MH	20.27 (3.86)	20.73 (2.22)	0.39	25.36 (2.90)	21.86 (2.48)	< 0.01*
HAMD	17.32 (6.07)	15.45 (5.29)	< 0.01*	10.55 (2.36)	10.95 (3.19)	0.12
HRV parameters						
MHR	75.66(11.02)	77.30(11.22)	0.14	79.27(14.90)	77.18(13.98)	0.02*
SDNN	22.01 (12.16)	22.25 (11.02)	0.83	16.07 (12.56)	17.79(11.54)	0.09
nLF	0.33 (1.92)	0.67 (1.77)	0.05*	-0.18 (1.76)	-0.63(1.75)	0.02*
nHF	0.82 (1.96)	1.37 (2.92)	0.09	-0.66 (1.48)	-0.29(1.25)	< 0.01*
LF/HF	1.58 (1.46)	1.80 (2.00)	0.58	2.98 (2.43)	1.76(1.78)	< 0.01*

Note: SD: standard deviation; PSQI: The Pittsburgh Sleep Quality Index; VT: Vitality Scale; MH: Menth Health Scale; HAMD: Hamilton Depression Rating Scale; MHR: Mean heart rate; SDNN: Standard deviation of all RR intervals; nLF: Normalized low frequency; nHF: Normalized high frequency

*p*-value: adopt paired t-test; *: *p* < 0.05

In the Sham group, significant differences in VT and MH were observed (*p* < 0.01), indicating worsened vitality and mental health post-intervention. Additionally, for HRV, significant differences in MHR, nLF, nHF, and LF/HF (*p* < 0.05) indicated a slower heartbeat, decreased sympathetic nerve activation, increased parasympathetic nerve activation, and a shift in the autonomic nervous system balance towards parasympathetic activity post-intervention.

### Post-intervention changes in PSQI, VT, MH, HAMD, and HRV parameters between the two groups

The ANCOVA results, adjusted for disability level, for changes in PSQI, VT, MH, HAMD, and HRV parameters between groups are presented in Table 3. Significant differences were observed in VT, MH, and HAMD (*p* < 0.05), indicating that the BM and RPS groups showed greater improvements in vitality, mental health, and depression levels post-intervention compared to the Sham group. Significant differences in the MHR and nLF (*p* < 0.05) indicated a significant increase in heartbeat and sympathetic nerve activation in the BM and RPS groups post-intervention.

**Table 3:** Changes of scores before and after the intervention in PSQI, VT, MH, HAMD, and HRV parameters between the two groups

	BBM and RPS group (*n* = 44)	Sham group (*n* = 44)	
Outcome variable	Mean (SD)	Mean (SD)	*p*-value
PSQI	0.08 (1.42)	-0.14 (2.03)	0.07
VT	1.27 (3.57)	-1.09 (2.26)	0.03*
MH	0.45 (3.45)	-3.50 (2.86)	< 0.01*
HAMD	-1.86 (2.31)	0.41 (1.72)	< 0.01*
HRV parameter			
MHR	1.64 (7.27)	-2.09 (5.86)	0.02*
SDNN	0.24 (7.11)	1.72 (5.84)	0.13
nLF	0.34 (1.09)	-0.45 (1.20)	0.01*
nHF	0.55 (2.07)	0.37 (0.91)	0.57
LF/HF	0.22 (2.63)	-1.22 (2.64)	0.17

Note: SD: standard deviation; PSQI: The Pittsburgh Sleep Quality Index; VT: Vitality Scale; MH: Menth Health Scale; HAMD: Hamilton Depression Rating Scale; MHR: Mean heart rate; SDNN: Standard deviation of all RR intervals; nLF: Normalized low frequency; nHF: Normalized high frequency

*p*-value: adopt ANCOVA adjusted for level of disability; *: *p* < 0.05

## Discussion

This study stands out as one of the few to combine audiovisual stimulation interventions for older adults with depressive symptoms. The results indicated that participants in the BBM and RPS groups showed significant improvements in vitality and depressive mood, with a notable increase in sympathetic nervous activity, while the Sham group experienced a significant decline in vitality and mental health, with a shift towards increased parasympathetic nervous activity. Furthermore, compared to the Sham group, the BBM and RPS groups demonstrated significantly greater improvements in vitality, mental health, and depression, along with a significant increase in heart rate and sympathetic nervous activity. This suggests that the BBM and RPS interventions have the potential to improve vitality, mental health, and depression in older adults with depressive symptoms in long-term care institutions.

The results in Table 2 suggest significant post-intervention improvements in vitality and depressive mood in the BBM and RPS groups, along with an increase in sympathetic nervous activity. This may be due to the modulatory effects of BBM and RPS on brain waves and neurotransmission. A meta-analysis study [30] found that BBM can influence memory, attention, anxiety, and pain perception without prior training, suggesting that BBM may improve mental health by modulating brain wave frequencies, thereby reducing anxiety and enhancing mood states, increasing vitality and alleviating depressive moods. Additionally, RPS may improve emotional regulation by modulating activity in specific brain regions, such as the amygdala and prefrontal cortex, which are closely related to emotion processing and mental health; for example, the default mode network (DMN) plays a key role in emotional regulation [31]. Increased HRV indicators reflect an increase in sympathetic nervous system activity, possibly due to the role of BBM and RPS in promoting brain activity and overall alertness [32].

The Sham group experienced significant post-intervention declines in vitality and mental health, along with a slower heart rate, decreased sympathetic nerve activation, and increased parasympathetic nerve activation (Table 2). This may be due to the lack of specific frequency stimulation (as in the BBM and RPS groups), leading to insufficient sympathetic nerve activity to produce positive mental health effects. Instead, it enhances parasympathetic activity, causing excessive relaxation, and thus reducing vitality [33]. The continued use of relaxation music alone can also lead to heightened parasympathetic activity, typically associated with rest and digestion but potentially detrimental to vitality in some cases (Thayer et al., 2012). The Sham group’s results underscore the crucial need for effective interventions beyond simple relaxation approaches to prevent further decline in older adults with depressive symptoms.

Comparing the BBM, RPS, and Sham groups in Table 3 after the intervention shows significant improvements in vitality, mental health, and depression, with a notable increase in sympathetic nervous activity. This could be due to the direct modulatory effects of BBM and RPS on brain function. BBM is believed to modify brain waves through the “frequency following response,” influencing emotions and cognitive functions [34]. Similarly, RPS, through rhythmic visual stimulation, may enhance brain alertness, thus affecting the autonomic nervous system, particularly the activity of the sympathetic nerves [35]. These modulatory effects can improve mental health, reduce depression, and increase physical vitality. Increased sympathetic nervous system activity in older adults with depressive symptoms can have both positive and negative effects. The sympathetic nervous system facilitates the body’s “fight or flight” response, and short-term activation can increase alertness and energy, potentially helping alleviate the low motivation and fatigue common in depressive symptoms [36]. However, prolonged increased sympathetic activity may lead to an excessive stress response, increasing the risk of cardiovascular problems, and potentially exacerbating anxiety symptoms [37]. Therefore, a careful balance of autonomic nervous system regulation is necessary in older adults with depression.

This study has several limitations that warrant consideration when interpreting its findings. First, recruitment from a single long-term care institution in central Taiwan limits the generalizability and applicability of the results to other populations. Second, the combination of BBM with relaxing music (including mask music) in this study may have confounded the isolated effects of BBM. Third, demographic variables, such as institutional atmosphere and interpersonal interactions, could have affected the participants’ depression or HRV parameters. Fourth, the short intervention period of only two weeks might not be sufficient to observe long-term mental health improvements or physiological changes. Fifth, the quasi-experimental design introduces the possibility of pre-existing differences between the control and experimental groups at baseline, potentially influencing the interpretation of the results. Future studies should consider a randomized controlled trial design. Despite these limitations, the results of this study provide an important reference for future interventions that use BBM combined with RPS to improve sleep quality, vitality, and depression in older adults.

## Conclusion

The results of this study indicate that combining BBM and RPS interventions in older adults with depressive symptoms in long-term care institutions significantly improves their vitality and mental health and reduces the severity of depression. Additionally, compared to the Sham group, this audiovisual stimulation intervention increased sympathetic nervous activity, suggesting potential positive effects on enhancing brain alertness and improving emotional states. However, these findings require further validation in studies with larger samples and longer durations. Future research should consider these preliminary results and conduct more clinical trials to explore the potential long-term effects of BBM and RPS in improving depressive symptoms in older adults.

## Data Availability

The datasets used and/or analyzed during the current study are available from the corresponding author on reasonable request.
